# Two-stage exchange Arthroplasty is a viable treatment for Periprosthetic joint infection in inflammatory diseases

**DOI:** 10.1186/s12891-020-03701-8

**Published:** 2020-10-14

**Authors:** Qiao Jiang, Jun Fu, Wei Chai, Li-Bo Hao, Yong-Gang Zhou, Chi Xu, Ji-Ying Chen

**Affiliations:** 1 Medical school of Chinese PLA, General Hospital of People’s Liberation Army, No.28 Fuxing Road, Haidian District, Beijing, 100853 China; 2 Department of Orthopedic Surgery, The first Medical Center, Chinese PLA General Hospital, Beijing, China; 3 Medical College, Nankai University, Tianjin, China

**Keywords:** Periprosthetic joint infection, Second-stage revision, Inflammatory diseases, Total joint arthroplasty

## Abstract

**Background:**

Inflammatory diseases are chronic autoimmune systemic autoimmune diseases, which may increase the risk of prosthetic joint infection (PJI) after total joint arthroplasty (TJA). However, to our best knowledge, few studies have studied the association between inflammatory diseases and subsequent failure after two-stage exchange reimplantation. The aims of this study were to identify the differences in (1) serum markers, synovial indicators and pathology results and (2) treatment outcomes following two-stage exchange arthroplasty between patients with or without inflammatory diseases.

**Methods:**

A retrospective review of 184 patients with PJI who underwent two-stage revision from 2014 to 2018 was conducted. PJI was diagnosed by using the MSIS criteria. Serum biomarkers, synovial fluid, organism and pathology results at the time of the PJI diagnosis and reimplantation were compared between patients with or without inflammatory diseases. Treatment success was defined according to the Delphi-based consensus criteria; Kaplan-Meier survivorship curves of the patients were generated and compared.

**Results:**

There was no difference in the biomarkers, pathology results or organism profile at the time of the PJI diagnosis. At reimplantation, the patients with inflammatory diseases generally had higher values of serum markers than those without inflammatory diseases. However, synovial white blood cell count was comparable in patients with inflammatory diseases (1142.8 ± 1385.3*10^9^/mL) and group C (1315.8 ± 1849.3*10^9^/mL, *p* = 0.841). The total treatment success rate was 91.3% (92% for individuals with inflammatory diseases and 91.2% for the controls). The survivorship of the inflammatory disease group was comparable with that of the control group.

**Conclusion:**

Two-stage exchange arthroplasty is a viable option for PJIs with inflammatory diseases. Synovial fluid analysis may be less affected by inflammatory diseases than serum markers did in the diagnosis persistent infection at reimplantation.

## Background

Inflammatory diseases are chronic systemic autoimmune diseases that mainly include rheumatoid arthritis (RA), psoriatic arthritis (PSA), juvenile idiopathic arthritis (JIA), ankylosing spondylitis (AS), and systemic lupus erythematosus (SLE). The prevalence of inflammatory diseases is rarely low, with a rate ranging from 0.05 to 1% in the general population [1]. Although several studies have suggested a decreasing trend in the occurrence of inflammatory diseases [2, 3], there are a considerable number of individuals with inflammatory diseases with severe arthritis who are candidates for total joint arthroplasty (TJA) [4].

Prosthetic joint infection (PJI) is a devastating complication that develops after TJA with an incidence of 1–3% following primary TJA and 3 to 5% following revision TJA [5–9], which leads to a tremendous burden for individual patients as well as the global health care industry [10]. Many studies have attempted to identify potential risk factors for PJI. For example, individuals with a medical history of an inflammatory disease have been shown to be independently associated with more than 4 times as many subsequent PJIs than those with osteoarthritis due to the long-term use of disease-modifying anti-rheumatic drugs (DMARDs) [11], biological drugs and corticosteroids [12–17].

The management of PJI remains challenging. Two-stage exchange arthroplasty remains the “gold standard” for chronic PJI in North America and East Asia [18, 19], with success rates ranging from 65 to 100% [20]. Due to the low incidence of inflammatory diseases, there are limited data on the outcomes following two-stage exchange arthroplasty for individuals with PJI and inflammatory diseases. In the clinic, surgeons frequently consider patients with inflammatory diseases to have inferior outcomes than those without inflammatory diseases. However, to the best of our knowledge, no study has been conducted to compare the outcomes between patients with or without inflammatory diseases. Additionally, whether the current thresholds of inflammatory biomarkers can be applied in patients with inflammatory diseases at PJI diagnosis remains unknown.

In this study, we investigated (1) whether there were any differences in the serum indicators, cultures and pathology results between patients with and without inflammatory diseases and (2) whether patients with inflammatory diseases have a poor prognosis after two-stage revision surgeries compared to those without inflammatory diseases.

## Methods

### Patients

We retrospectively reviewed the database of our hospital to identify all patients who underwent two-stage revision for PJI after total knee arthroplasty (TKA) or total hip arthroplasty (THA). A total of 226 patients (228 joints) underwent two-stage revision between 2014 and 2018, of which 28 patients (12.3%) suffered from inflammatory diseases (including rheumatoid arthritis, psoriatic arthritis and ankylosing spondylitis). The diagnoses of inflammatory diseases were made on the basis of the Assessment of SpondyloArthritis International Society’s criteria for AS [21], 2010 Rheumatoid Arthritis Classification Criteria for RA [22], and criteria proposed by Taylor for psoriatic arthritis [23]. All inflammatory disease patients underwent medical therapy involving DMARDs, biological drugs or corticosteroids in the rheumatology department, and the inflammatory diseases were not active or showed low levels of activity before surgery. In addition, non-biologic DMARDs continued to be used to control the underlying inflammatory diseases, while biologic DMARDs were withheld perioperatively and restarted after evidence of wound healing after reimplantation.

The exclusion criteria were as follows:1. incomplete medical recordings at reimplantation, including serological tests and culture results; 2. patients with less than 1- year follow-up or no infection occurrence within this period. PJI was diagnosed based on the MSIS criteria, and patients who did not meet the MSIS criteria were excluded. In addition, we excluded 6 patients who underwent spacer exchange rather than reimplantation in two-stage procedure because an identical organism was isolated from at least two preoperative synovial fluids. After implementing the aforementioned criteria, a total of 184 patients were included in the final analyses; of these, 25 patients were diagnosed with inflammatory diseases, including 13 with rheumatoid arthritis, 8 with ankylosing spondylitis and 4 with psoriatic arthritis.

The medical records were reviewed manually in detail to determine whether there were any differences between patients with and without inflammatory diseases in the demographic data (sex, age, body mass index [BMI], and type of joint [knees or hips]) or American Society of Anesthesiologists (ASA) score. We included comorbidities as defined by the international consensus on PJI [24] and other risk factors, including hepatitis and cardiovascular disease [25]. The serologic test results (including erythrocyte sedimentation rate [ESR], C-reactive protein [CRP], interleukin-6 [IL-6], fibrinogen, d-dimer), pathology results, and organism culture test results were compared between the two groups at the time of resection and reimplantation. The resistant organisms were defined as methicillin-resistant *Staphylococcus aureus* (MRSA), methicillin-resistant *Staphylococcus epidermidis* (MRSE) and vancomycin-resistant enterococcus (VRE).

### Treatment protocol

An institutional standard protocol for two-stage procedures was performed. During the first stage procedure, the infected prosthesis was removed, and then thorough debridement and irrigation was performed. Synovial fluid and multiple tissue specimens were routinely taken for microbial and histological analysis intraoperatively. An antibiotic-loaded spacer was inserted which contained a total of 6 g of meropenem and vancomycin per forty grams of methyl-methacrylate cement polymer, and the formulation of the two antibiotics is adjusted according to the type of isolated bacteria. For fungal infections, an additional 200 mg of voriconazole was added to the bone cement. Post the resection, all patients received at least 6 weeks of systematic antibiotics basing on culture sensitivity reports and institutional guidelines.

Prior to reimplantation, joint aspiration was performed for patients with clinical suspicion after at least 2-week “antibiotic holiday”. The determination of proper timing to perform reimplantation was based on the combination of laboratory test, the improvement in clinical symptoms and synovial analysis. During reimplantation, the antibiotic-loaded cement spacer was removed, and then sterile saline water was used to irrigate. 3–5 samples were acquired and sent to microbial culture. Furthermore, three to five additional tissue samples were obtained intraoperatively and sent for frozen sectioning. A positive histopathology result was defined as more than 5 polymorphonuclear neutrophils (PMNs) per × 400 high-power field (HPF) in at least five HPFs [26]. After reimplantation, intravenous second generation cephalosporins were prescribed after surgery until the results of the intraoperative culture were negative. For patients with one or more positive cultures during reimplantation, 6–10 weeks antibiotics were prescribed, including 2–4 weeks of IV antibiotics followed by 4–6 weeks of oral antibiotics.

The median interval between the 1st stage and 2nd stage was 126.00 (25.00 to 1203.00) days in the non-inflammatory disease group and 126.00 (37.00 to 391.00) days in the inflammatory disease group (*P* = 0.998).

### Definition of the endpoints and treatment success

Primary endpoints for this study were defined as follows: 1. Recurrence of infection resulting in spacer exchange; 2. Reinfection (according to MSIS criteria) after reimplantation; 3. long-term antibiotic suppression at the time of the last follow-up; and 4. death related to PJI.

We determined treatment success using the following Delphi-based consensus criteria [27, 28]: 1. infection eradication, characterized by a healed wound without fistula, drainage, pain, or infection recurrence caused by the same organism strain; 2. no subsequent surgical intervention for infection after reimplantation surgery; and 3. no occurrence of PJI-related mortality.

### Statistical analysis

All of the statistical analyses were performed with the statistical software package R (http://www.R-project.org, The R Foundation). The categorical data were summarized as the absolute value and percentage. The continuous data are presented as the mean and standard deviation (SD). The demographic and clinical characteristics were compared between groups with the Student’s *t*-test if the data were normally distributed; if the data were not normally distributed, the Mann-Whitney test was used for continuous variables and the chi-square test or Fisher’s exact test was used for categorical variables. Kaplan-Meier survivorship curves with treatment failure as an endpoint were generated. Differences in survivorship between patients with or without inflammatory diseases were assessed using the log-rank test. A retrospective power analysis was calculated based on the 5-year survival rates. A *p*-value less than 0.05 was considered significant.

## Results

### General information

The patient characteristics are shown in Table 1. There was no significant difference in age, sex, ASA, type of surgery, joint function score, or smoking habits between the inflammatory disease and non-inflammatory disease groups. Interestingly, patients in the inflammatory disease group had a higher prevalence of renal disease than the control group (20.00% VS 2.52%, *p* = 0.003), while there was no significant difference in the other relevant risk factors according to our analysis. More alcohol abusers were found in the inflammatory diseases group (28.00% VS 8.18%, *p* = 0.009), indicating the need for more effective management.

**Table 1:** Patients data and demographics

	Non-inflammatory diseases(*n* = 159)	Inflammatory diseases (*n* = 25)	*P*-value
Patient characteristics			
Mean age, years (SD)	57.12 (14.70)	55.96 (14.77)	0.737
Mean BMI, kg/m^2^ (SD)	25.26 (3.74)	23.49 (4.17)	0.031
Mean ASA (SD)	2.08 (0.41)	2.12 (0.33)	0.608
Gender, n (%)			0.740
Female	77 (48.43)	13 (52.00)	
Male	82 (51.57)	12 (48.00)	
Joint, n (%)			0.283
Knee	69 (43.40)	8 (32.00)	
Hip	90 (56.60)	17 (68.00)	
Comorbidities, n (%)			
Diabetes	25 (15.72%)	2 (8.00%)	0.541
Hepatitis	6 (3.77%)	1 (4.00%)	1.000
Cardiovascular	16 (10.06%)	2 (8.00%)	1.000
Malignancy	8 (5.03%)	0 (0.00%)	0.601
Renal	4 (2.52%)	5 (20.00%)	0.003
Alcohol abuse	13 (8.18%)	7 (28.00%)	0.009
Mean joint function score (SD)	40.85 (17.41)	36.52 (17.99)	0.323

*BMI* body mass index, *ASA* American society of anesthesiologists; joint function score, measured by The Hospital for Special Surgery (HSS) score for knee and Harris score for hip

### Lab, microbiology and pathology tests at spacer insertion

We retrospectively collected and analysed all data from the database on the lab, culture and pathology tests for both groups at the time of spacer insertion. The results are shown in Table 2. No significant differences were found in the lab test results. However, all inflammatory indicators were elevated according to the MSIS criteria. In the non-inflammatory disease group, we mainly found CNS (*n* = 42, 26.42%), *S. aureus* (*n* = 33, 20.75%), gram-negative bacillus (*n* = 12, 7.55%) and other pathogens. 35 out of 129 microorganisms were resistant to methicillin and vancomycin. In the inflammatory disease group, we mainly found CNS (*n* = 5, 20.00%), *S. aureus* (*n* = 3, 12.00%), gram-negative bacillus (*n* = 2, 8.00%) and other pathogens. 2 out of 14 microorganisms were resistant to methicillin. The positive pathology rate was 62.77% in the non-inflammatory disease group and 73.68% in the inflammatory disease group.

**Table 2:** Lab tests and culture results among patients with and without inflammatory diseases at spacer insertion

	Non-inflammatory diseases(*n* = 159)	Inflammatory diseases(*n* = 25)	*P*-value
Serum biomarkers, mean (SD)			
CRP (mg/l)	29.9 (33.4)	23.3 (22.4)	0.552
IL-6 (pg/ml)	30.03 (97.49)	22.54 (34.22)	0.991
ESR, (mm/h)	44.34 (26.76)	38.27 (22.14)	0.370
Fibrinogen, (g/l)	5.05 (1.30)	4.83 (1.12)	0.164
D-dimer, (g/ml)	1.92 (1.44)	1.23 (0.64)	0.106
Mean Synovial WBC, 10^9^/ml (SD)	23,994.78 (30,090.00)	20,571.67 (34,426.31)	0.406
Microbiology results, n (%)			
*Staphylococcus aureus*	33 (20.75)	3 (12.00)	0.286
Coagulase negative Staphylococcus	42 (26.42)	5 (20.00)	0.373
*Enterococcus faecalis*	9 (5.66)	1 (4.00)	1.000
Streptococcus	5 (3.14)	1 (4.00)	0.577
Gram negative bacillus	12 (7.55)	2 (8.00)	1.000
Fungus	11 (6.92)	0 (0.00)	0.364
Polymicrobial organisms	12 (7.55)	2 (8.00)	1.000
Other organisms	5 (3.14)	0 (0.00)	1.000
Positive pathology *, n (%)	100 (62.77)	18 (73.68)	0.753
Sinus tract, n (%)	43 (27.22)	7 (28.00)	1.000

*ESR* erythrocyte sedimentation rate, *CRP* c-reactive protein, *IL-6* interleukin-6; positive pathology, more than 5 polymorphonuclear neutrophils (PMNs) per ×400 high-power field (HPF) in at least five HPFs

### Microbiology and pathology tests at Reimplantation

Table 3 shows the microbiology and pathology test results at reimplantation for the two groups. There were two patients had ≥2 positive cultures in inflammatory disease group, including 1 (4.17%) Gram-negative bacillus and 1 (4.17%) CNS which was resistant to methicillin. In addition, 18 of 159 cases with ≥2 positive cultures were identified in the non-inflammatory diseases group. We mainly found gram-negative bacillus (*n* = 7, 4.40%), *Staphylococcus aureus* (*n* = 3, 1.89%), CNS (*n* = 1, 1.26%), fugus (*n* = 2, 1.26%) and other organisms (*n* = 5, 3.14%). 2 out of 18 organisms were resistant to methicillin. There were 3 (10.52%) patients with positive frozen section in inflammatory diseases group versus 30 (18.95%) patients in control group (*p* = 0.520). 6 (3.77%) patients in non-inflammatory group had sinus tract communicating with prosthesis.

**Table 3:** Culture results and frozen section among patients with and without inflammatory diseases at reimplantation

	Non-inflammatory diseases(*n* = 159)	Inflammatory diseases(*n* = 25)	*P*-value
Microbiology results, n (%)			
Staphylococcus aureus	3 (1.89)	0 (0.00)	1.000
Gram negative bacillus	7 (4.40)	1 (4.17)	1.000
CNS	1 (1.26)	1 (4.17)	0.143
Fungus	2 (1.26)	0 (0.00)	1.000
Other organisms	5 (3.14)	0 (0.00)	1.000
Positive Pathology*, n (%)	30 (18.95)	3 (10.52)	0.570
Sinus tract, n (%)	6 (3.77)	0 (0.00)	1.000

### Patients’ follow-up

According to the Delphi-based consensus criteria, treatment failure occurred in 14 (8.8%) patients in the non-inflammatory disease group, including 12 patients who underwent further surgery and 2 patients death related to PJI. In patients with inflammatory diseases, treatment failure occurred in 2 (8.0%) patients including 1 patient underwent further surgery and 1 patient with long-term antibiotic suppression. In addition, there were 7 (9.09%) patients failed after TKA and 9 (8.41%) patients failed after THA (*p* = 0.872). The mean duration of follow-up was 33.9 months (15.9 to 51.9) in the non-inflammatory disease group and 35.5 months (16.2 to 54.8) in the inflammatory disease group (Table 4). No significant difference in the joint function score was observed in the PJI without inflammatory disease group or PJI with inflammatory disease group (*p* = 0.084).

**Table 4:** Outcomes of patients with and without inflammatory diseases

	Non-inflammatory disease(*n* = 159)	Inflammatory diseases (*n* = 25)	*P*-value
Mean follow-up, months (SD)	33.9 (18.0)	35.5 (19.3)	0.704
Failure, n (%)	14 (8.8)	2 (8.0)	1.000
Mean joint function score (SD)	83.92 (10.89)	76.16 (19.26)	0.084

Joint function score, measured by The Hospital for Special Surgery (HSS) score for knee and Harris score for hip

**Fig. 1:**
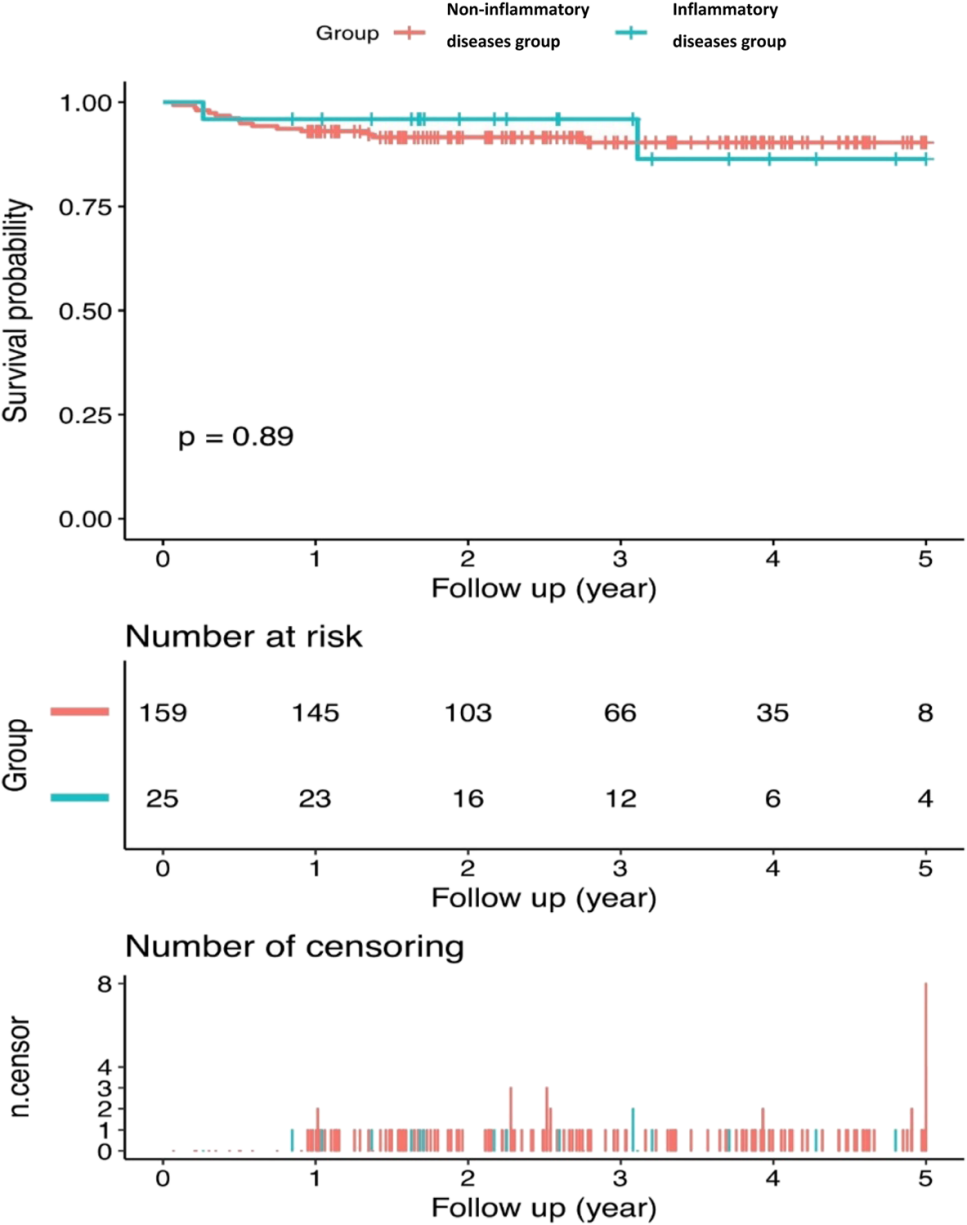
Kaplan-Meier survival curve regarding treatment failure in patients with and without inflammatory diseases

Overall, the survivorship of the individuals free from PJI in the non-inflammatory disease group was 93.08% (95% CI, 89.22 to 97.11%) at 1 year and 90.39% (95% CI, 85.58 to 95.47%) at 5 years, and the survivorship of the individuals free from PJI in the inflammatory disease group was 96.00% (95% CI, 88.62 to 100.00%) at 1 year and 86.40% (95% CI, 69.23 to 100.00%) at 5 years (Fig. 1). No significant difference was found between the two groups in the probability of survival (*p* = 0.89).

### Comparison of lab tests at reimplantation

Based on our follow-up results, since there were patients with persistent infection in both groups, the laboratory tests at reimplantation may be affected by both the inflammatory diseases and underlying infection. To eliminate the effects of persistent infection, we divided patients into three groups: Group A: reinfection patients with or without inflammatory diseases (*n* = 16); Group B: cured patients with inflammatory diseases (*n* = 23); Group C: cured patients without inflammatory diseases (*n* = 145). The mean values of serum tests and synovial WBC were compared among three groups. In general, inflammatory diseases, as well as the persistent infection, increased the value of serum markers. There were no significant differences in values of any serum markers (CRP, ESR, IL-6, CRP and D-dimer) between group A and group B. In addition, patients in group A and group B had significant higher serum tests than patients in group C. However, inflammatory diseases may have less effect in the synovial WBC. Patients in group A had higher mean level of synovial WBC (3320.4 ± 1633.9) than patients in group B (1142.8 ± 1385.3, *p* = 0.023) and group C (1315.8 ± 1849.3, *p* = 0.026). The mean level of synovial WBC was comparable in group B (1142.8 ± 1385.3) and group C (1315.8 ± 1849.3, *p* = 0.841). The details of mean value of serum tests and synovial WBC in three groups were shown in Table 5.

**Table 5:** Lab tests in reinfection group, inflammatory group and non-inflammatory group at reimplantation

	Group	Mean (SD) values	Group	Mean (SD) values	*P*-value
CRP (mg/l)	A	25.5 (44.0)	B	17.9 (16.5)	0.456
	A	25.5 (44.0)	C	6.1 (7.9)	< 0.001
	B	17.9 (16.5)	C	6.1 (7.9)	0.002
IL-6 (pg/ml)	A	20.9 (27.6)	B	23.4 (40.3)	0.835
	A	20.9 (27.6)	C	5.1 (5.3)	< 0.001
	B	23.4 (40.3)	C	5.1 (5.3)	< 0.001
ESR (mm/hr)	A	23.2 (23.2)	B	27.5 (21.9)	0.292
	A	23.2 (23.2)	C	13.7 (12.7)	0.011
	B	27.5 (21.9)	C	13.7 (12.7)	0.007
Fibrinogen (g/l)	A	4.1 (1.2)	B	4.2 (1.0)	0.650
	A	4.1 (1.2)	C	3.4 (0.8)	0.002
	B	4.2 (1.0)	C	3.4 (0.8)	0.001
D-dimer (ug/ml)	A	1.9 (1.8)	B	2.8 (3.1)	0.846
	A	1.9 (1.8)	C	1.7 (1.3)	0.660
	B	2.8 (3.1)	C	1.7 (1.3)	0.035
Mean synovial WBC, 10^9^/ml	A	3320.4 (1633.9)	B	1142.8 (1385.3)	0.023
	A	3320.4 (1633.9)	C	1315.8 (1849.3)	0.026
	B	1142.8 (1385.3)	C	1315.8 (1849.3)	0.841

## Discussion

Prosthetic joint infection (PJI) is a devastating complication, and inflammatory diseases have been reported to be an important risk factor for PJI in many articles [11–14]. The diagnosis of PJI depends on the combination of the culture results, lab test results, clinical symptoms and pathology results. Classic serological markers, including ESR and CRP, are widely used in diagnoses, and serum fibrinogen was suggested to be useful in diagnoses in a study by LI R et al. [29]. Many articles have researched the utility of lab, culture and pathology tests in diagnosing PJI prior to two-stage revision [30–32]. However, to the best of our knowledge, no studies have compared the differences in the indicator values, culture test results and pathologies between patients with and without inflammatory diseases, mainly because of the rarity of inflammatory diseases in individuals with PJI.

According to our research, inflammatory disease patients show no significant difference in the serum biomarkers or synovial WBC count at the time of spacer insertion. The average ESR, CRP and synovial WBC count values are all above the thresholds in the MSIS criteria, indicating that the MSIS criteria are also optional standards for diagnosing inflammatory disease patients. The average fibrinogen levels in both groups were higher than 4.10 g/l, which is in accordance with the results reported by LI R et al. [29], suggesting that fibrinogen is a helpful indicator for both patients with and without inflammatory diseases. Coagulase-negative staphylococci were the most common organisms in the inflammatory disease group in our research, while no significant difference was found in the type or ratio of microorganisms. An excessive focus on the elevation of the inflammatory indictors caused by the inflammatory diseases was shown to be unnecessary, as the infection activated the immune system and led to even higher levels of the biomarkers in individuals with well-controlled inflammatory diseases.

At the reimplantation stage, there is no gold standard for the diagnosis of PJI, and the MSIS criteria are considered to have low sensitivity because of long-term antibiotic suppression [32]. However, we found a significant difference between the non-inflammatory disease and inflammatory disease groups in the serum markers. The average levels of serum markers were clearly elevated, even though the inflammatory diseases of all patients included in the analysis showed low levels of activity. The synovial WBC count showed good consistency between the two groups and was less affected by the immune changes caused by the inflammatory diseases. Many articles have explained the elevation in serum and synovial IL-6, ESR, and CRP in patients with inflammatory diseases [33, 34], causing ultrahigh sensitivity in diagnosing PJI before insertion. Significant differences in the inflammatory indicators in the inflammatory disease group were observed in our research, which may sometimes mislead clinical doctors and make it difficult to distinguish inflammatory disease related PJIs using serum biomarkers alone. In addition, fibrinogen recommended by LI R et al. [29] failed to distinguish inflammatory diseases and PJIs at reimplantation due to its elevation in both inflammatory diseases and PJIs. However, synovial fluid is a local immune response that is less influenced by inflammatory diseases. The recommended marker is the synovial WBC count at the time of reimplantation because of its good agreement between patients with and without inflammatory diseases. XIE K et al. [31] reported that the synovial IL-6 level showed higher sensitivity and specificity than the serum IL-6 level in diagnosing PJI, but more research is required to determine whether the synovial IL-6 level is less affected by inflammatory diseases. More research is needed to identify the thresholds of synovial indicators for diagnosing PJIs in individuals with inflammatory diseases and PJIs.

Two-stage revision is widely reported to be a viable procedure for prosthetic joint infection [10, 35, 36], and patients with inflammatory diseases suffer from a higher risk of infections than those without inflammatory diseases [1, 2]. However, we found that inflammatory diseases and PJIs can be resolved, and the survivorship in individuals with PJIs and inflammatory diseases was as high as 86.4% (95% CI, 69.2 to 100%) in our hospital. Regarding joint function, apparent improvement was observed between the perioperative and postoperative procedures. More than two-stage revision, one-stage revision was suggested to be a reliable treatment. In a study of 85 patients underwent one-stage revision, the 10-year infection-free survival was 94% and the surgery-free survival was 75.9% [37]. However, there were limited studies reported the treatment outcome of one-stage revision in patients with inflammatory diseases. In a study involved 200 RA patients with, the survival rate for patients underwent debridement and retention of components (DAIR), two-stage revision, and resection arthroplasty was 32, 79, and 62%, after 5-year follow-up [38]. DAIR had 5.9 times increased risk of treatment failure when compared to two-stage revision [39]. More studies are needed to compare the treatment outcomes of different surgical options in patients with inflammatory diseases.

There were several limitations in our research. First, this was a retrospective study, and certain biases of retrospective studies cannot be avoided. Although we reviewed most cases that were documented, some errors may exist. Second, a limitation of the study is the small number of patients with inflammatory diseases and PJIs. Therefore, we did not divide the patients into rheumatoid arthritis, psoriasis and ankylosing spondylitis groups to analyse them separately, which may have resulted in an underpowered study. In addition, the inflammatory disease group in this study only consisted of RA, AS and PAS patients, while patients with other conditions, such as systemic lupus erythematosus, need to be studied further. Third, all inflammatory disease patients in our research received medical treatment to control the inflammatory diseases, and no active inflammatory diseases were visible. The level of activity of the inflammatory diseases may affect the failure rate and lab indicators, which should be confirmed in future studies. Fourth, there were some patients without joint fluid in our study. However, according to the guideline of American Academy of Orthopaedic Surgeons, the presence of “dry tap” is allowed and routine joint aspiration is not recommended. Some laboratory tests, such as synovial alpha-defensin and synovial CRP, were not the routine examination in our hospital, so whether these tests are also affected by inflammatory diseases are still unclear. Moreover, all patients in our study underwent two-stage revision. Other treatment protocol, such as one-stage revision and DAIR, may be also viable for PJIs with inflammatory diseases, Further studies are needed to compare the different treatment options. Finally, we continue using the non-biologic DMARDs to control the underlying inflammatory diseases, which may lead to certain bias in serum tests and histopathology findings.

## Conclusion

Two-stage revision is a viable option in PJIs with or without inflammatory diseases. Patients with inflammatory diseases had significant higher levels of serum markers than patients without inflammatory diseases, which may lead to misdiagnosis of persistent PJI. However, synovial WBC was comparable in patients with and without inflammatory diseases.

## Data Availability

We do not wish to share our data, because some of the patient’s data regarding individual privacy, and according to the policy of our hospital, the data could not be shared with others without permission.

## Abbreviations

RA: Rheumatoid arthritis
PSA: Psoriatic arthritis
JIA: Juvenile idiopathic arthritis
AS: Ankylosing spondylitis
SLE: Systemic lupus erythematosus
TJA: Total joint arthroplasty
PJI: Prosthetic joint infection
DMARDs: Disease-modifying anti-rheumatic drugs
TKA: Total knee arthroplasty
THA: Total hip arthroplasty
BMI: Body mass index
MSIS: Musculoskeletal Infection Society
ASA: American Society of Anesthesiologists
ESR: Erythrocyte sedimentation rate
CRP: C-reactive protein
IL-6: Interleukin-6
WBC: White blood cell
SD: Standard deviation
PMN: Polymorphonuclear
CNS: Coagulase negative staphylococci
HSS: The hospital for special surgery knee score
